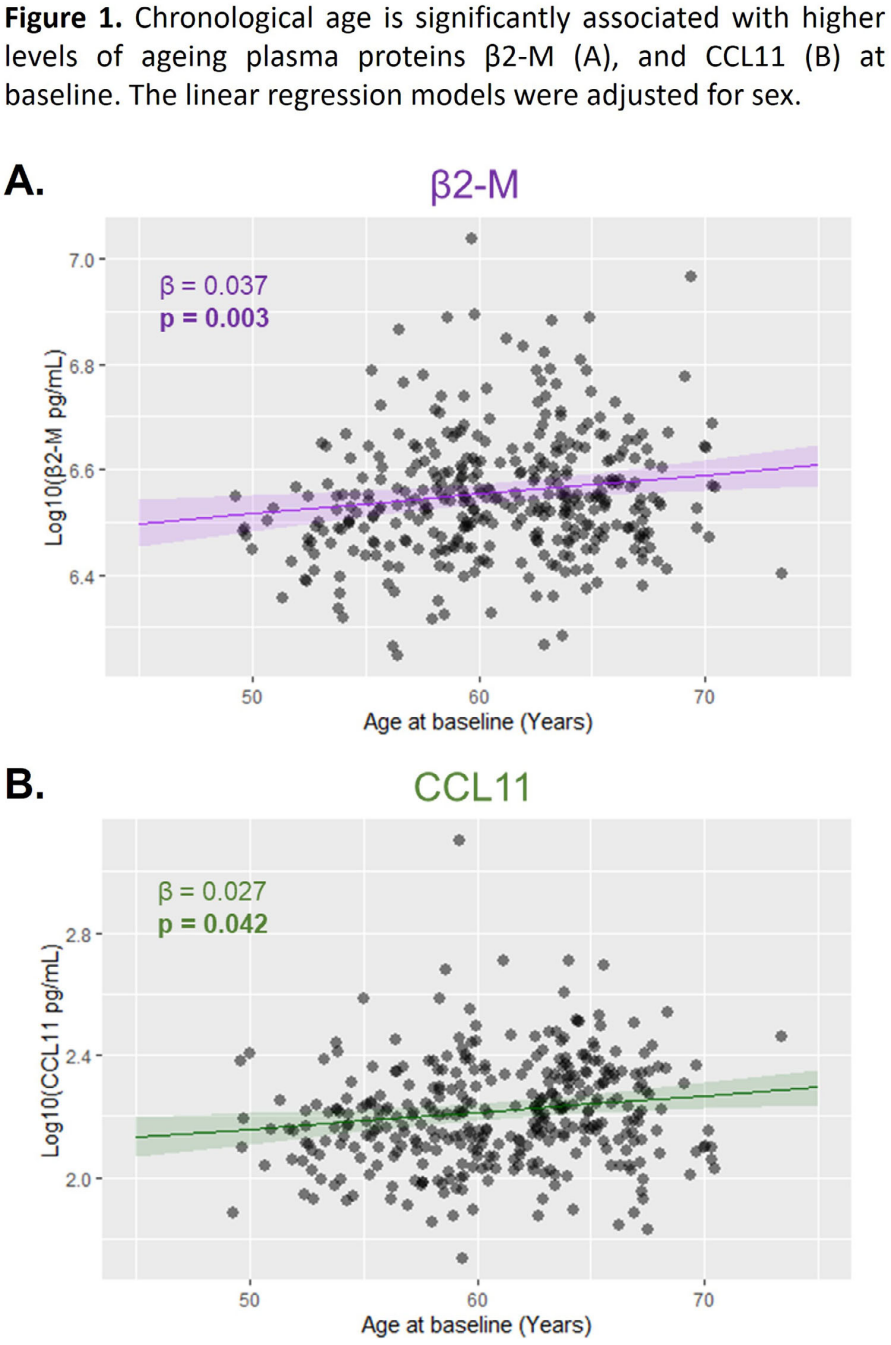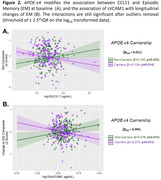# APOE‐ε4 carriership modifies the association of CCL11 and sVCAM1 plasma protein levels with cognitive performance in individuals at risk of Alzheimer's disease

**DOI:** 10.1002/alz.091646

**Published:** 2025-01-09

**Authors:** Luis Felipe Hernández‐Villamizar, Pol Segura‐Retana, Armand González Escalante, Marta Milà‐Alomà, Paula Ortiz‐Romero, Marina De Diego‐Osaba, Luisa Braun‐Wolhfahrt, David López‐Martos, Gonzalo Sánchez‐Benavides, Henrik Zetterberg, Kaj Blennow, Federica Anastasi, Marc Suarez‐Calvet

**Affiliations:** ^1^ Barcelonaβeta Brain Research Center (BBRC), Pasqual Maragall Foundation, Barcelona Spain; ^2^ Hospital del Mar Research Institute (IMIM), Barcelona Spain; ^3^ Departament de Bioquímica i Biologia Molecular, Universitat Autònoma de Barcelona (UAB), Barcelona, Catalonia Spain; ^4^ Universitat Politècnica de Catalunya (UPC), Barcelona Spain; ^5^ Universitat Pompeu Fabra, Barcelona Spain; ^6^ Centro de Investigación Biomédica en Red de Fragilidad y Envejecimiento Saludable (CIBERFES), Madrid Spain; ^7^ Universiteit Maastricht, Maastricht Netherlands; ^8^ Universitat de Barcelona (UB), Barcelona Spain; ^9^ Clinical Neurochemistry Laboratory, Sahlgrenska University Hospital, Mölndal Sweden; ^10^ Department of Psychiatry and Neurochemistry, Institute of Neuroscience and Physiology, University of Gothenburg, Mölndal Sweden; ^11^ Department of Molecular Neuroscience, UCL Institute of Neurology, London United Kingdom; ^12^ UK Dementia Research Institute at UCL, London United Kingdom; ^13^ Centre for Genomic Regulation (CRG), Barcelona Institute of Science and Technology (BIST), Barcelona Spain; ^14^ Servei de Neurologia, Hospital del Mar, Barcelona Spain

## Abstract

**Background:**

In murine models, peripheral blood factors have been identified as having either a brain rejuvenating or ageing effect. However, it is unclear whether these blood factors have similar effects in humans. We aimed at testing the association between these blood factors and cognitive performance in cognitively unimpaired (CU) individuals at risk of Alzheimer’s disease (AD).

**Method:**

We included 414 CU participants from the ALFA+ cohort [mean age: 61.1(±4.72), women:60.4%, APOE‐ε4 carriers:54.8%, and amyloid‐positive:34.2%]. A subset of participants (n=341) had longitudinal follow‐up [mean follow of 3.28 years (0.50)]. Amyloid positivity was determined by CSF‐Aβ42/40<0.071. Blood factors associated with an ageing effect [β2‐microglobulin(β2‐M), CCL2, CCL11, CCL19, Haptoglobin, sVCAM1] or rejuvenating effect (CSF2 and TIMP2) were measured using ELISA, MSD, or Simoa immunoassays.

Through linear regression analyses we first tested the association between chronological age and each protein, correcting by sex. Then, we tested the association of each protein with baseline cognition and longitudinal cognitive changes at follow‐up, adjusting for age, sex and years of education. Cognitive data included the PACC score and domain‐specific composites (attention, episodic memory[EM] and executive function). The modifying effect of sex, APOE‐ε4 carriership, and amyloid status was also tested and significant interactions stratified.

**Result:**

β2‐M and CCL11 significantly increased with chronological age (Figure 1). Only APOE‐ε4 carriership modified the association of age‐related blood factors CCL11 (p_int_=0.011) and sVCAM1 (p_int_=0.006) with cross‐sectional and longitudinal EM, respectively (Figure 2). In APOE‐ε4 non‐carriers, higher CCL11 was associated with better EM at baseline (β=0.105, p=0.025), while higher sVCAM1 was associated with better EM at follow‐up (β=0.076, p=0.010). Inversively, in APOE‐ε4 carriers, higher CCL11 was associated with worse EM at baseline (β=‐0.104, p=0.014), and higher sVCAM1 was associated with worse EM at follow‐up (β=‐0.073, p=0.014).

**Conclusion:**

We observe an association between higher CCL11 and sVCAM1 and worse cognition in APOE‐ε4 carriers. This may suggest that the APOE‐ε4 carriers are more susceptible to the ageing effect of these blood factors.